# In memoriam –Scott Federhen (October 21, 1952 - May 5, 2016)

**DOI:** 10.1186/s40793-016-0160-z

**Published:** 2016-06-08

**Authors:** Conrad L. Schoch, Ilene Karsch-Mizrachi, James M. Ostell

**Affiliations:** National Center for Biotechnology Information, National Library of Medicine, National Institutes of Health, Building 45, 8600 Rockville Pike, Bethesda, MD 20894 USA

Scott (Herbert) Federhen passed away on May 5, 2016, after a short illness. Scott was well known for heading the National Center for Biotechnology Information’s (NCBI’s) Taxonomy project, which he started in 1991. Under his leadership it became an international standard for nomenclature and classification.

Born in 1952, Scott obtained a MS degree in Computer Science, focused on denotational semantics of AI languages, from University of Maryland in 1980. An MS in Biology from MIT followed, with a focus on control structures in biological systems. He joined NCBI in 1990, shortly after its establishment. His dual training in both Computer Sciences and Biology served him well as he oversaw tremendous growth in the NCBI Taxonomy Database, developed numerous submission and database enhancements, and collaborated regularly with many other NCBI groups and members of the International Nucleotide Sequence Database Collaboration (INSDC). The INSDC continues to follow the NCBI Taxonomy Database classification.

Under Scott’s guidance, the NCBI Taxonomy group was occasionally involved in scientific controversy, when underlying molecular data supported taxonomic changes. One example is the early inclusion of birds (Aves) under the Dinosauria in the NCBI classification. Scott also spearheaded NCBI’s efforts with the Barcode of Life project, an international initiative to identify and archive the barcode (a short genetic sequence from a standard part of the genome) for every species of life.

Recently, Scott was instrumental in greatly expanding the annotation of type material in the NCBI taxonomic database. This allowed for improved taxonomic accuracy. Almost a year to the day before his death, he presented a proposal he developed for a groundbreaking change in how taxonomic names would be treated at NCBI. His sly sense of humor is evidenced in the title: “A Modest Proposal for making the Genomes of GenBank beneficial to the Publick, and preventing them from being a Burthen to the Curators and Taxonomists” (a nod to Johnathan Swift’s famous satire). The group he invited to the workshop included members of the International Committee on Systematics of Prokaryotes (ICSP) and the Judicial Commission on Prokaryotic Nomenclature, as well as bioinformaticians and taxonomists working with genomic similarity measures. Scott proposed a set of measures to represent a scaffold of reliably identified sequences that could be used to find and correct misidentified genomes. The invitees also agreed on a set of principles that will guide future efforts. It is anticipated that this project will continue and expand, but its inception was a testament to Scott’s drive and vision.

Scott Federhen will be remembered as an adventurous eater, kind colleague and devoted Red Sox fan with an exceptionally broad intellectual range. He leaves behind his wife, Sheree, a daughter, Erica, a son, Luke, and many friends.

